# Sonographic imaging features of alveolar soft part sarcoma: Case series and literature review

**DOI:** 10.1097/MD.0000000000031905

**Published:** 2022-11-18

**Authors:** Wenxue Li, Shanshan Zhang, Wenting Fan, Diancheng Li, Hui Tian, Dongdong Che, Lei Yu, Shuang Gao, Yiqun Liu

**Affiliations:** a Department of Ultrasound, Peking University People’s Hospital, Beijing, People’s Republic of China.

**Keywords:** alveolar soft part sarcoma, excision, MRI, sonographic

## Abstract

Alveolar soft part sarcoma (ASPS) is a rare tumor but potentially fatal condition. Understanding the imaging and clinical features of ASPS is of certain value for preoperative qualitative diagnosis and clinical treatment of tumors. Nevertheless, there have been only 11 documented case reports describing the sonographic features in the English literature.

**Methods:**

Three patients with confirmed ASPS occurring primarily in the limbs were enrolled in this study. Complete surgical excision was performed with conservative limb function. We pay particular attention to the ultrasonographic features and performed a literature review of ASPS cases.

**Results:**

With regular surveillance, one patient had no symptom recurrence and two developed lung and/or breast metastasis later. The specific sonographic findings were heterogeneous hypoechoic, well-circumscribed, and lobulated or round contours on grayscale images, abundant flow signals of intratumoral and extratumoral tubular structures on color Doppler images.

**Conclusion subsections:**

Its low incidence rate and lack of characteristic clinical manifestations often result in misdiagnosis of ASPS. The specific sonographic findings may add useful diagnostic information.

## 1. Introduction

Alveolar soft part sarcoma (ASPS) is a rare tumor that was first defined and named by Christopherson et al in 1952.^[1]^ It is known to comprise 0.2% to 0.9% of all soft tissue sarcomas and tends to occur between ages 15 and 35 years.^[2,3]^ It predominantly occurs in the lower extremities, and it has been described in a variety of unusual locations, including the urinary bladder, breast, larynx, uterine cervix and bone.^[4,5]^ Understanding the imaging and clinical features of ASPS is of certain value for preoperative qualitative diagnosis and clinical treatment of tumors. Nevertheless, there have been a few reports describing the sonographic features of ASPS. To our knowledge, only 11 cases of the ultrasound features in ASPS were published and available on PubMed. Here, we report 3 cases of ASPS occurring primarily in the limbs, pay particular attention to the ultrasonographic features and review the literature. The written informed consents were obtained from the patients for publication of this case report and accompanying images.

### 1.1. Case 1

A 30-year-old male was referred to the Department of Orthopedics, for evaluation of painless swelling of the left arm with a period of 1 week. Upon palpation, the patient presented a 10 cm long mass. The mass appears ill-defined, without cutaneous retraction, medial hardness or mobility in the transverse plane.

**Figure 1.**
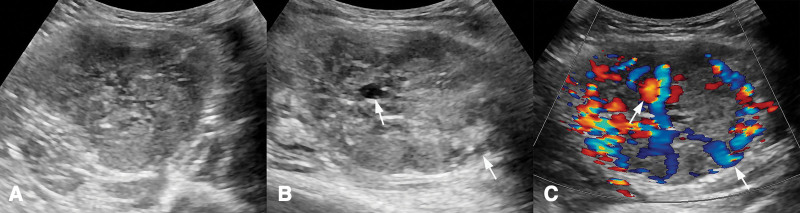
Real-time ultrasound images of the triceps brachii muscle tumor. (A) Sonogram shows a 7.9 × 6.1 × 4.8 cm, well limited, heterogeneous hypoechoic tumor in the triceps brachii muscle. (B) Tube-like echoless is evident in central and peripheral of the tumor (arrows). (C) Color Doppler ultrasound shows abundant blood flow signals in intratumoral and extratumoral areas, and the tubular structures are extremely hypervascularized (arrows).

**Figure 2.**
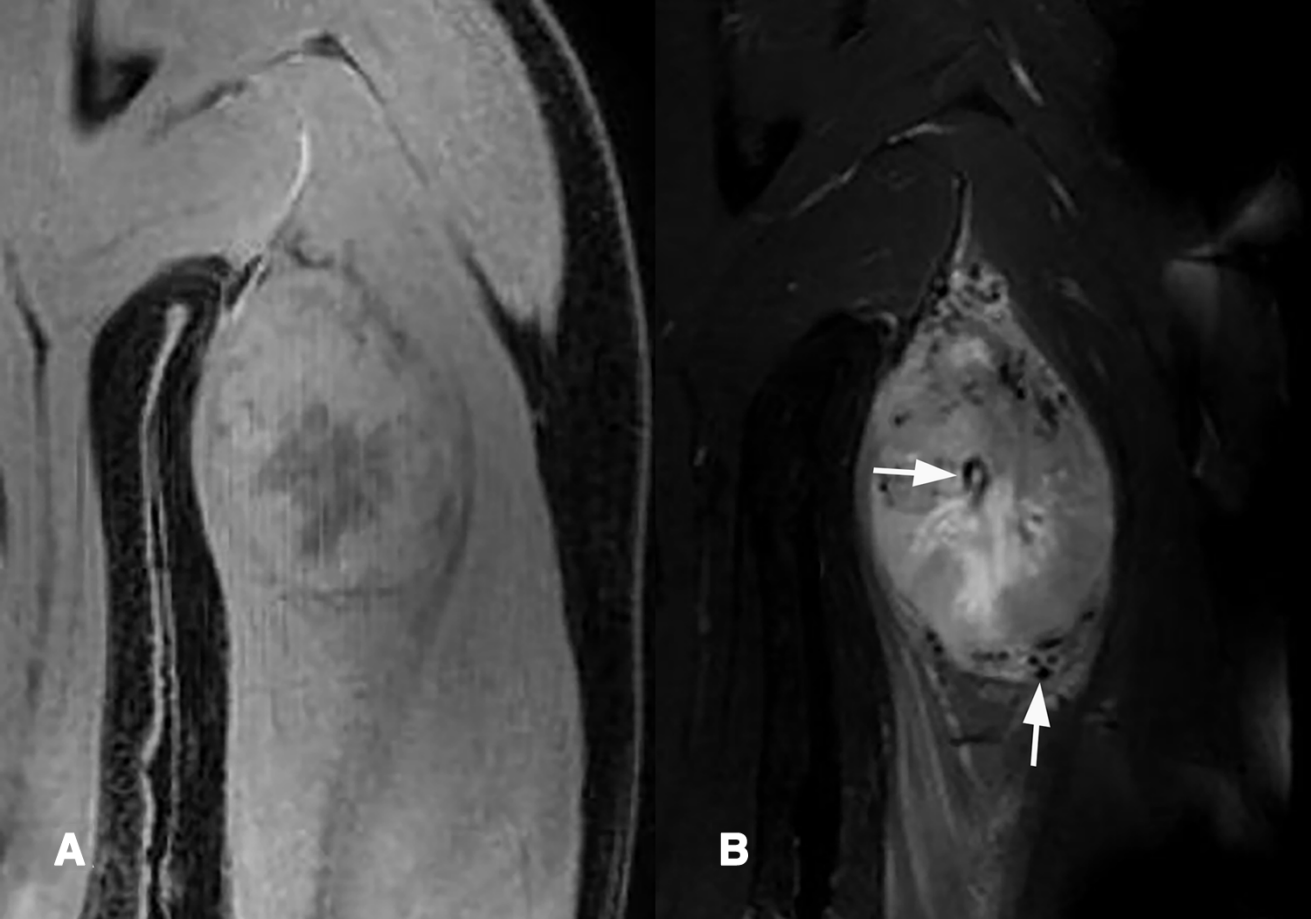
Preoperative MRI of an alveolar soft part sarcoma in the triceps brachii muscle. (A) Coronal T1-weighted image shows an moderate-signal intensity. (B) Coronal T2-weighted fat-suppressed image shows heterogeneously hyperintense and had flow voids that were both central and peripheral (arrows). MRI = magnetic resonance imaging.

Ultrasonography of the mass was performed using a real-time convex array scanner of 5 MHz and a linear array scanner at a nominal frequency of 12 MHz (HI VISION Ascendus Ultrasound System, Hitachi Medical Systems, Japan) with the patient in the sitting position. The lesion was heterogeneous hypoechoic tissue measuring 7.9 × 6.1 × 4.8 cm in the triceps brachii muscle, well limited, and with tube-like echoless in the center and periphery of the tumor. Color Doppler ultrasound showed indiscriminate color flow signals in anechoic area (Fig. 1). The artery flow spectrum was detected by pulse wave Doppler. Computed tomography (CT) and magnetic resonance imaging (MRI) scanning of the right upper extremity were performed using real-time helical thin slice CT scanning and 3.0‑Tesla MRI scanning. MR studies demonstrated a large lesion in the triceps brachii muscle. It appeared well circumscribed on MRI and had moderate signal intensity on T1-weighted imaging. It was heterogeneously hyperintense at T2-weighted imaging and had flow voids that were both central and peripheral (Fig. 2). The lesion demonstrated slightly high density without significant bony destruction on CT.

An ultrasound-guided biopsy with pathology analysis revealed a distinctive alveolar pattern with large, round to polygonal tumor cells with eosinophilic cytoplasm. Pathology then confirmed that the lesion was an ASPS owing to positive TFE3 (transcription factor E3) staining.

Metastatic examination included brain MRI and CT of his chest, abdomen and pelvis, and no masses or lesions were found. Complete surgical excision was performed with conservative limb function. Pathological analysis revealed a pseudoalveolar pattern and sinusoidal vessels, and the immunohistochemistry results demonstrated diffuse nuclear immunoreactivity to TFE3 and vimentin, which confirmed the initial hypothesis of ASPS. The clinical course was favorable in the short and medium term with good wound healing.

Four months after diagnosis, multiple masses in both lungs had developed. Contrast-enhanced CT imaging of lungs showed mild homogeneously enhancing lobulated lesions in the bilateral lobs (left approximately 0.5 cm and right approximately 0.6 cm) with clear and smooth borders. Right-sided larger lung lesions underwent total gross excision. Histopathology of lung lesions was suggestive of metastatic deposits of ASPS. He was given 3 courses of chemotherapy with vincristine, ifosfamide, and cisplatin. On follow-up ultrasound imaging, there was no evidence of local relapse on the left arm 13 months after surgery.

### 1.2. Case 2

A 30-year-old female presented with a history of swelling in her left thigh for approximately 1 and a half years. She visited her doctor because of the increased size of the lesion and recurrent numbness sensation. Physical examination revealed a well demarcated, non-tender mass on the left thigh.

**Figure 3.**
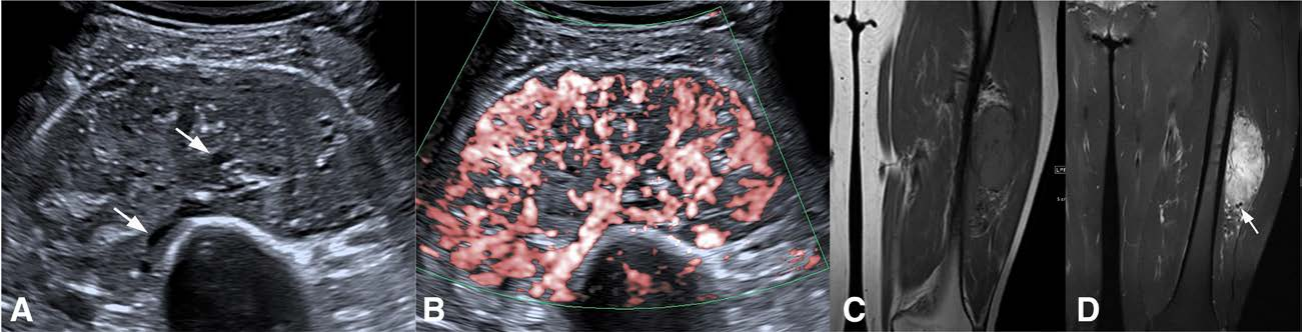
Sonography and MRI of the left thigh. (A) Gray scale ultrasound reveals a heterogeneous hypoechoic tumor with clear boundary in the vastus intermedius muscle, well limited, and with tube-like echoless in it (arrows). (B) Color Doppler ultrasound reveals marked increased vascularity in the tumor, and the tubular structures are hypervascularized. (C) MRI reveals long T1 abnormal signals in the vastus intermedius muscle. (D) T2-weighted coronal view with fat saturation shows hyper intensity, and flow voids in peripheral and interior region of the tumor(arrows). MRI = magnetic resonance imaging.

Ultrasonography of the mass was performed using a real-time convex array probe of 6 MHz (Aploi i800 Ultrasound System, Canon Medical Systems, Japan) with the patient in the supine position. Grayscale ultrasound revealed a heterogeneous hypoechoic tissue measuring 8.2 × 8.2 × 5.5 cm with a clear boundary in the vastus intermedius muscle that was well limited, and had tube-like echoless (Fig. 3A). Color Doppler flow imaging revealed markedly increased vascularity in the center and periphery of the tumor, and the tubular structures were extremely hypervascularized (Fig. 3B). MRI revealed long T1 and T2 abnormal signals in the vastus intermedius muscle. Tumor parenchyma was markedly enhanced after enhancement scanning. Some flow voids in the peripheral and interior regions of the tumor were also noted (Fig. 3C and D).

A diagnosis of ASPS was rendered after ultrasound-guided biopsy. Immunohistochemistry then confirmed this because of positive TFE3. CT chest showed multiple bilateral lung metastases before surgery. This patient underwent medial femoral muscle resection under general anesthesia. She was discharged in good condition 12 days after the operation. On follow-up imaging, there was no evidence of local disease on the left thigh. Ultrasonography revealed multiple breast metastases 14 months after surgery. The patient declined chemotherapy and radiation throughout her postoperative course, and she passed away from disease-related complications 38 months later.

### 1.3. Case 3

A 23-year-old female presented to the hospital with a slight pain mass in the left thigh. The mass was first noticed by the patient 2 years earlier and presented with a relatively indolent clinical course. Before 1 month, she presented with intermittent pain and discomfort on the lateral side of the left thigh. Physical examination revealed an ovoid, immobile mass with a longitudinal diameter of 9 cm and some tenderness on palpation.

**Figure 4.**
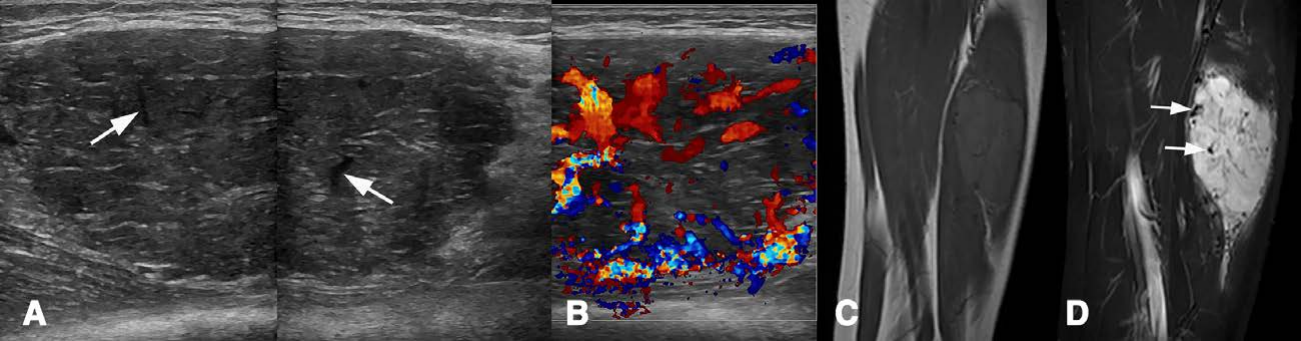
Sonography and MRI of the left thigh. (A) Gray scale ultrasound reveals a heterogeneous hypoechoic mass lesion in the vastus lateralis muscle, well limited, and with tube-like echoless in it (arrows). (B) Superb microvascular imaging shows abundant color flow signals in anechoic area in central and peripheral of the tumor. (C) Coronal T2-weighted fat-suppressed image shows high signal lesion in the vastus lateralis muscle, and multiple flow voids appeared both central and peripheral (arrows). MRI = magnetic resonance imaging.

Ultrasonography of the mass was performed using a 9 MHz real-time linear array probe (Logiq E9 Ultrasound System, GE Medical Systems, America) with the patient in the lateral position. The lesion was heterogeneous hypoechoic tissue measuring 8.3 × 5.1 × 4.0 cm in the vastus lateralis muscle, well limited, and with tube-like echoless (Fig. 4A). Superb microvascular imaging showed abundant color flow signals in anechoic area in the center and periphery of the tumor (Fig. 4B). Contrast-enhanced MRI findings showed an oval low T1WI signal and high T2WI signal lesion in the vastus lateralis muscle, the edge of the lesion appeared to be shallow lobulated, and the boundary between local and adjacent muscle tissue was not clear. The internal signal was not uniform, and multiple flow voids appeared both central and peripheral (Fig. 4C and D).

Histological examination demonstrated ASPS by ultrasound-guided biopsy. No signs of metastasis were found before the operation. Therefore, wide resection of the tumor was performed. Primary wound closure was achieved. On follow-up imaging, there was no evidence of local disease 5 months after surgery.

## 2. Literature review

The following Medline terms were searched in different combinations: “sarcoma, alveolar soft part,” “ultrasonography,” “ultrasonics,” and “diagnostic imaging.” Original articles, case reports, and case series on adult patients published in English between 1980 and January 2020 were critically analyzed. Full texts and references from relevant papers were reviewed to identify additional data sources. We identified 13 articles including 13 case reports. Eleven of 13 cases ultrasonic found are well described. Clinical and imaging features are summarized in Table 1. Two additional cases of sonographic details were not available.^[17,18]^

**Table 1:** Ultrasonic characteristics of our case series and the previously reported 11 cases of alveolar soft part sarcoma.

Authors	Age/gender of patients	Location	Ultrasonic features				Treatment	Follow-up
			Size	Main echo	Boundary	Flow signals		
Present case 1	30/M	Left upper extremity	7.9 × 6.1 × 4.8 cm	Hypoechoic, heterogeneously	Well-circumscribed	Hypervascularity, tubular structures intratumor and peritumor with rich blood perfusion	Wide excision, chemotherapy	Multiple metastases to lungs 4 mo later
Present case 2	30/F	Left lower extremity	8.2 × 8.2 × 5.5 cm	Hypoechoic, heterogeneously	Well-circumscribed	Hypervascularity, tubular structures intratumor and peritumor with rich blood perfusion	Wide excision	Multiple metastases to lungs before surgery and to breasts 14 mo later
Present case 3	23/F	Left lower extremity	8.3 × 5.1 × 4.0 cm	Hypoechoic, heterogeneously	Well-circumscribed	Hypervascularity, tubular structures intratumor and peritumor with rich blood perfusion	Wide excision	NSR 5-mo
Tsu-Te Liu et al^[6]^ (1997, China)	20/F	Pectoris major muscle	3 cm	Hypoechoic, heterogeneously	Well-circumscribed	Hypervascularity	Wide excision	Unknown
Mahul B Amin et al^[7]^ (2006, USA)	25/F	Bladder	2.5 × 2.3 cm	Hypoechoic	Well-circumscribed	Unknown	Excision	Urethral recurrence
Yi-Chen Lai et al^[8]^ (2009, China)	19/F	Right lower extremity	6.3 × 4 × 3 cm	Hypoechoic	Well-circumscribed	Hypervascularity, low RI, tubular structures intratumor with rich blood perfusion	Wide excision	Unknown
A. M. Abdoulwahab et al^[9]^ (2017, Niger)	60/M	Right upper extremity	15 cm	Hypoechoic, heterogeneous,	Well-circumscribed	Hypervascularity,	Monoblock surgical excision, chemotherapy	Unknown
Linli Qiu et al^[10]^ (2017, China)	3/M	Penis	1.8 × 1.6 × 2.1 cm	Hypoechoic, heterogeneous	Well-circumscribed	Hypervascularity	Organ-sparing penectomy, hemotherapy	NSR 28-mo
Rumeal D. Whaley et al^[11]^ (2019, USA)	71/M	Thyroid	5.7 × 4.5 × 4.4 cm	Hypoechoic, heterogeneous	Well-circumscribed	Hypervascularity	Thyroid lobectomy	NSR 5-mo
Bin Wang et al^[12]^ (2020, China)	6/F	Right lower extremity	2.3 cm	Hypoechoic heterogeneous	Well-circumscribed	Hypervascularity	Enlarged resection, radiotherapy	NSR 3-yr
N N Hanna et al^[13]^ (1996, USA)	26/F	Metastases to the breast	1.2 cm, 1.5 cm	Hypoechoic, homogeneous	Well-circumscribed	Unknown	Chemotherapy	Multiple metastases to breasts and lungs 2 yrs later
Hyo Soon Lim et al^[14]^ (2006, Korea)	29/F	Metastases to the breast	1.4 cm	Hypoechoic, homogeneous	Well-circumscribed, posterior sonic enhancement	Hypervascularity, enlarged vessels around the tumor and dilated intratumor vessels at the peripheral portion	Wide excision, chemotherapy	Unknown
Andrea Madrigrano et al^[15]^ (2008, USA)	18/F	Metastases to the breast	3.8 × 2.1cm	Hypoechoicand heterogeneous	Well-circumscribed	Unknown	Lumpectomy	Unknown
Yuka Asano et al^[16]^ (2019, Japan)	11/F	Metastases to the breast	2.2 × 1.6 × 2.1 cm	Hypoechoic, heterogeneous	Well-circumscribed	Hypervascularity	Wide excision	Unknown

Of the 14 patients, including our 3 case reports, ten were female and 4 male, and ages ranged from 3 to 71 years (median 24 years). Patients with ASPS generally range from 20 to 30 years, and women are more likely to develop tumors than men.^[19,20]^ Ten cases located the primary tumor with ultrasonography examination. The tumors occurred in the pectoris (n = 1), upper extremity (n = 2), lower extremity (n = 4), penis (n = 1), bladder (n = 1) and thyroid (n = 1). ASPS is most commonly found in the extremities,^[21]^ which corresponds with our case series. The remaining 4 cases were diagnosed with breast metastasis by using high-frequency ultrasound. The length-diameter of the ASPS tumor ranged from 15 mm to 150 mm (average, 49.9 mm). The measurements of limb tumors were relatively larger than those of primary ASPS in other sites (average, 80.0 mm to 32.5 mm). In our cases, 1 patient had lung metastases at diagnosis and 2 developed lung and/or breast metastasis later. The clinical features of ASPS are slow growth, high rate of early hematogenous metastasis, late local recurrence and fatality. Metastasis may already exist at the initial clinical manifestation.^[13,19]^

## 3. Discussion

ASPS usually presents as a slowly growing, painless soft tissue mass and rarely causes functional impairment. Unlike the majority of sarcomas, ASPS has high frequency of metastases, primarily to the lungs, brain and bones.^[19]^ Therefore, it is usually first diagnosed by symptoms that metastasize to the lungs and brain.^[22]^ The 5-year overall survival rate of patients with metastasis can decline sharply.^[4]^

Ultrasound is essential for determining extent of the lesion, which helps in evaluating resectability, and for most tumors complete resection is intricately related to prognosis. After a diagnostic biopsy, all patients but 1 received curative surgical resections. For ASPS, the most common pattern of internal echogenicity includes hypoechoic areas with well-defined boundaries, and these masses may be heterogeneous or homogeneous and vary with size. Two reported cases with a diameter less than 15 mm showed homogeneous echo, and the others with larger sizes were described as heterogeneous. Tumor margins with well-circumscribed and lobulated or round contours were also frequently observed. It has been hypothesized that hypoecho may be attributed to the tumor growth pattern. Histologically, ASPS is characterized by a uniform nest‑like pattern of large, rounded or polygonal tumor cells with eosinophilic granularity, a pseudoalveolar appearance, and often central necrosis.^[23]^

Ultrasound also characterizes vascularity. All cases showed hypervascularity with color Doppler ultrasound owing to its abundant blood supply and well vascularized.^[24]^ The intratumoral sinusoidal vessels showed dense hyperplasia, fusion and sometimes expansion.^[25]^ The intratumoral vessels could be detected by high-frequency ultrasound. Owing to its hypervascularity, ASPS might be confused with an arteriovenous malformation.^[26]^ In 1 case, color Doppler ultrasound revealed very low RI, approximately 0.24 to 0.58. Low RI indicated the presence of a direct shunt between the artery and dilated vein.^[8]^

In addition, our cases indicated additional imaging features: the appearance of tubular structures, which is in consistent with 2 other cases describing the vascular nature of this tumor.^[8,14]^ Color Doppler ultrasound shows that these tubular structures are abundant colored flows. This finding has been reported to be a feature of this tumor and it has been described to exist at both the center and periphery of the tumor. It corresponds to intratumoral and peritumoral flow voids on MRI.^[27]^ The surrounding veins often twisted and dilated, occasionally showing mural attenuation disproportionate to the lumen diameter. This vascular change may represent early sinus vascular remodeling, which may later be incorporated into the tumor.^[25]^

Metastases that occur primarily in mammary tissue are considered extremely rare.^[15]^ In our cases, the size of the breast tumors was relatively small, approximately 22.2 mm. The most common ultrasonographic appearance of breast metastasis is 1 or more well-circumscribed, homogeneous or heterogeneous hypoechoic nodules,^[13,15,16]^ which cannot be differentiated from benign masses from a purely radiological point of view. Although the breast is an unusual metastatic site, ultrasonographic evidence of breast nodules may be the initial or later finding of alveolar soft tissue sarcoma. Therefore, breast nodules should be considered metastatic diseases in patients with extramammary alveolar soft tissue sarcoma. In 1 case, the lesions showed enlarged vessels around the tumor and dilated intratumor vessels at the peripheral portion on grayscale ultrasonic imaging, and there were abundant color flow signals on color Doppler ultrasound.^[14]^ These imaging manifestations are similar to those of primary ASPS but are not common in benign lesions, which indicates the vascular pathological feature of the tumor.

Despite the rarity of ASPS, we reviewed the sonographic characteristics of a case series. This enables a more comprehensive evaluation of the imaging features of this tumor than before. The sonographic features add useful diagnostic information of ASPS and will aid surgeons in performing wide surgical resection to reduce the risk of local recurrence. A diagnosis of ASPS should be considered when a solid tumor arising in muscle is heterogeneous hypoechoic, well-circumscribed, and lobulated or round contours on grayscale images, hypervascularity on color Doppler images. Additional diagnostic features that we identified are the presence of intratumoral and extratumoral tubular structures, which indicate large vessels at both the center and periphery of the tumor. Prominent intratumoral and extratumoral vessels of a mammary nodule, which are similar to primary tumors, may be a sign of breast metastasis, especially in a young woman with a history of ASPS. In the future, the direct correlation between imaging features and anatomical and pathological results will further deepen our understanding of the imaging manifestations of this rare and unique tumor.

## Author contributions

**Conceptualization:** Shanshan Zhang.

**Data curation:** Wenting Fan, Diancheng Li, Hui Tian, Dongdong Che, Lei Yu, Shuang Gao.

**Formal analysis:** Wenxue Li.

**Funding acquisition:** Yiqun Liu.

**Investigation:** Yiqun Liu.

**Methodology:** Yiqun Liu.

**Supervision:** Yiqun Liu.

**Writing – original draft:** Wenxue Li.

**Writing – review & editing:** Yiqun Liu.
